# Rhabdomyosarcoma of prostate presenting as bladder outlet obstruction in a young adult

**DOI:** 10.3332/ecancer.2013.360

**Published:** 2013-10-02

**Authors:** Pranab Prabhakaran, Rajitha Sanjayan, Thara Somanathan, Geetha Narayanan

**Affiliations:** 1 Department of Medical Oncology, Regional Cancer Centre, Thiruvananthapuram, 695011 Kerala, India; 2 Department of Pathology, Regional Cancer Centre, Thiruvananthapuram, 695011 Kerala, India

**Keywords:** bladder outlet obstruction, prostate, rhabdomyosarcoma

## Abstract

A 19-year-old boy presented with bladder outlet obstruction and on evaluation was found to have prostatomegaly, which on biopsy was diagnostic of embryonal rhabdomyosarcoma (RMS). He had pulmonary metastasis and received chemotherapy with cyclophosphamide, doxorubicin, vincristine, actinomycin D, and radical radiotherapy. At one year, his prostatic tumour has resolved completely. Embryonal RMS of prostate occurs more commonly in infancy and childhood, so occurrence in young adults is rare, and a high index of suspicion is essential for early diagnosis and treatment.

## Introduction

Bladder outlet obstruction (BOO) in a young male is an uncommon symptom and is often benign in aetiology. Rhabdomyosarcoma (RMS) of the prostate in young adults is rare, and very few cases are reported in the literature [1]. We present a case of RMS prostate in a young adult presenting as BOO.

## Case Report

**Figure 1.**
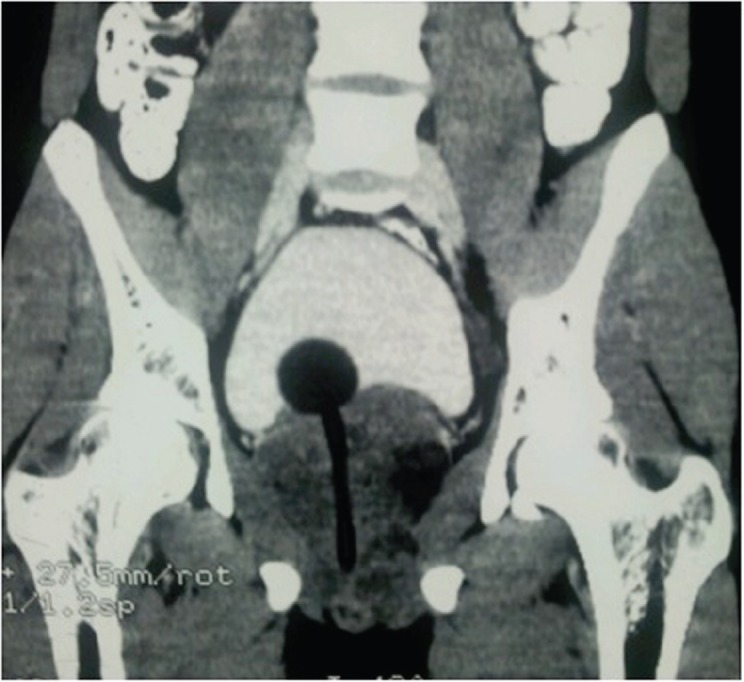
CT scan of the pelvis showing heterogeneously enhancing mass replacing the prostate infiltrating the bladder base and Foley’s bulb and catheter *in situ*.

A 19-year-old boy presented with one month’s history of dysuria and increased frequency of urination followed by acute urinary retention. He had no fever or weight loss or any history of high-risk behaviour. On examination, he had grade 3 prostatomegaly with no lymphadenopathy or hepatosplenomegaly. Blood counts, urine microscopy, and renal and liver function tests were normal. Serum lactate dehydrogenase level was 715 U/L, serum alkaline phosphatase and prostate specific antigen (PSA) levels were normal. A computed tomography (CT) scan of the abdomen and pelvis revealed a 9 × 7 × 6.5 cm^3^ heterogeneously enhancing mass replacing the prostate with infiltration to the bladder base reaching inferiorly up to the left ischiorectal fossa (Figure 1). There were no calcifications within and no lymph nodes.

**Figures 2.**
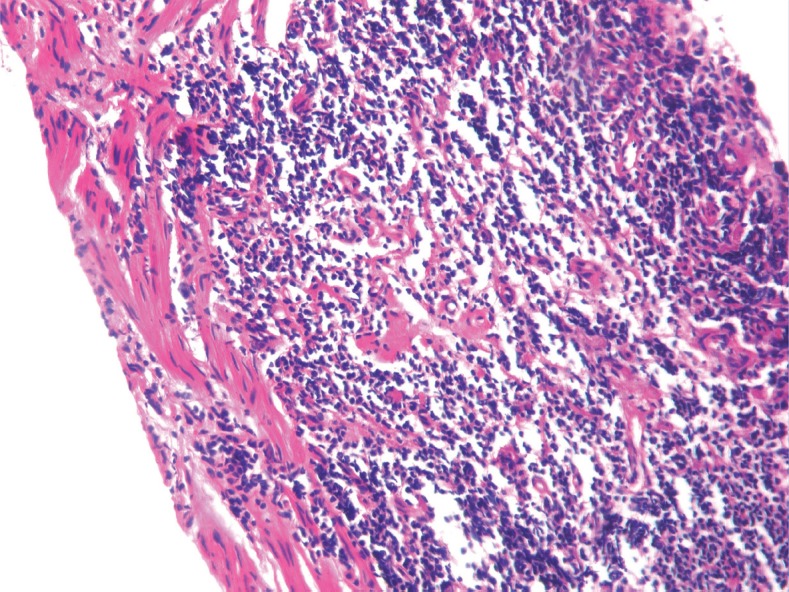
Hematoxylin and eosin (H & E) stained image showing sheets of cells having hyperchromatic nuclei at 10x magnification.

**Figures 3.**
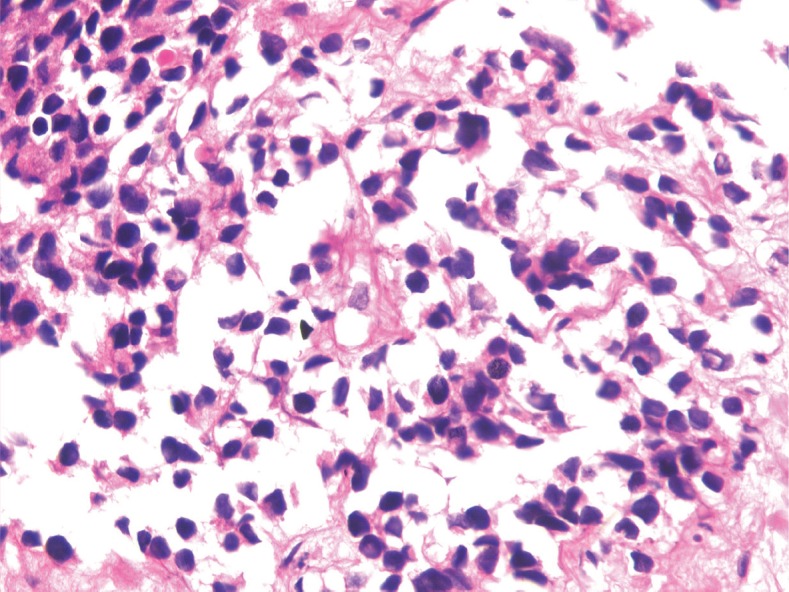
H & E stained image showing sheets of cells having moderate amount of eosinophilic cytoplasm and hyperchromatic nuclei at 40x magnification.

**Figure 4.**
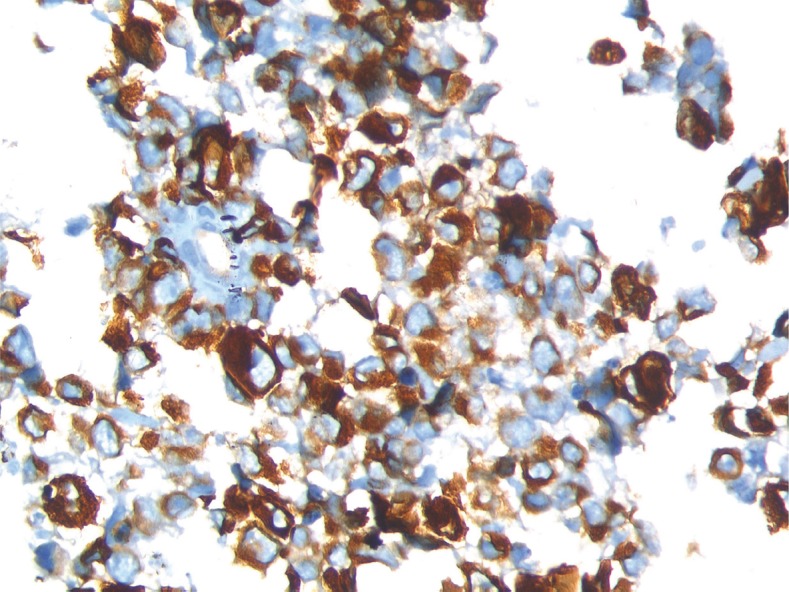
Immunohistochemistry 40x image showing desmin positivity.

**Figure 5.**
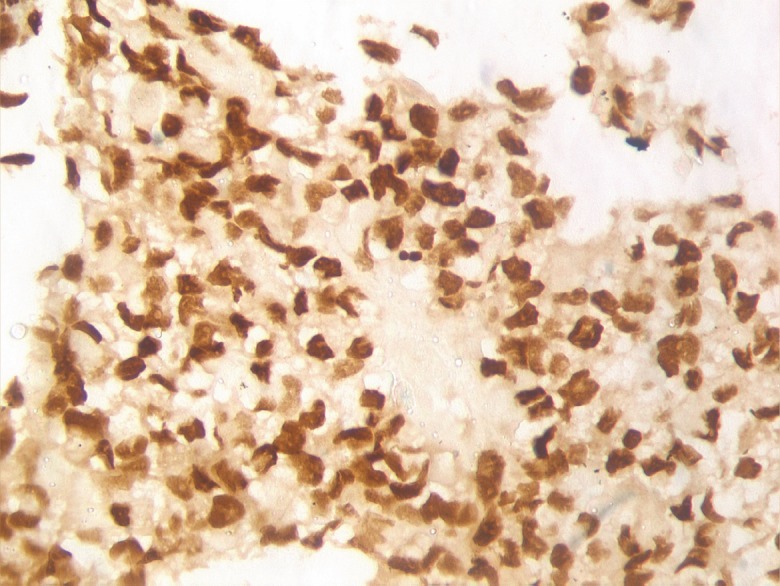
Immunohistochemistry 40x image showing myogenin positivity.

A needle biopsy from the prostate showed sheets of cells having a moderate amount of eosinophilic cytoplasm and hyperchromatic nuclei (Figures 2 and 3). On immunohistochemistry, the tissue specimen was positive for desmin and myogenin (Figures 4 and 5) and negative for cytokeratin and leucocyte common antigen (CD45), suggestive of embryonal RMS. A CT of the chest showed well-defined nodular densities scattered in the left lung, suggestive of pulmonary metastasis. There was no bony metastasis, and bone marrow was normal.

The patient was started on chemotherapy with cyclophosphamide, doxorubicin, vincristine, and actinomycin D. After three months of chemotherapy, the lung nodules disappeared along with tumour shrinkage. He received local radiotherapy (XRT) (50 Gy/28#). At ten months, he developed haemorrhagic cystitis, which improved with supportive measures. A repeated CT scan of the pelvis one year after initiating chemotherapy showed a normal prostate.

## Discussion

RMS of the prostate occurs predominantly in male infants and children and is a highly malignant tumour. Very few cases have been reported in patients above 18 years [1, 2]. It mostly presents with symptoms of dysuria or urinary obstruction. It is characterised by rapid growth, and the consequent local invasion leads to symptoms of BOO or rectal compression. The lungs, liver, and skeleton are the main sites for metastases. CT is helpful in characterising the primary tumour and in detecting spread to regional lymph nodes [3]. The prostatic acid phosphates and PSA levels are usually normal. The diagnosis is made on transrectal needle biopsy or transurethral resection or biopsy specimens [2].

RMS is histologically divided into embryonal, alveolar, and pleomorphic subtypes. Immunohistochemistry shows positivity for desmin and skeletal muscle markers, which include MyoD1, myogenin, sarcomeric actin, and myoglobin. Recently, cytoplasmic staining with WT1 has been added as an immunomarker for rhabdomyoblastic differentiation [4].

Bladder preservation is the major goal of therapy for young patients with tumours arising in the bladder and/or prostate. Patients with a primary tumour of the bladder/prostate causing outlet obstruction are usually treated with XRT following initial chemotherapy to relieve BOO. Presently, a more effective chemotherapy and XRT have increased the frequency of bladder salvage. For patients with residual tumour following chemotherapy and XRT, appropriate surgical management may include partial cystectomy and prostatectomy [5, 6].

## Conclusions

BOO in a young male is an unusual symptom. A high index of suspicion alone will help in detecting RMS of the prostate before it is too late.
